# TULP4 degrades RYBP to enhance DNA damage repair and chemosensitivity of pancreatic ductal adenocarcinoma

**DOI:** 10.1016/j.gendis.2024.101288

**Published:** 2024-04-05

**Authors:** Jingyao Dai, Su Liu, Yangyang Xie, Yixuan Wang, Xiyun Bian, Tian Yu, Tiantian Li, Linchuang Jia, Zhigang Zhao, Zhiqiang Liu, Xiaozhi Liu

**Affiliations:** a Hepatobiliary Surgery Department, Air Force Medical Center, Beijing 100142, China; b Tianjin Medical University Cancer Institute and Hospital, National Clinical Research Center for Cancer, Tianjin Key Laboratory of Cancer Prevention and Therapy, Tianjin's Clinical Research Center for Cancer, Tianjin 300060, China; c Department of Pathophysiology, School of Basic Medical Science, Tianjin Medical University, Tianjin 300060, China; d Tianjin Key Laboratory of Epigenetics for Organ Development in Preterm Infants, Tianjin Fifth Central Hospital, Tianjin 300450, China; e Department of Medical Oncology, Tianjin First Central Hospital, School of Medicine, Nankai University, Tianjin 300192, China

Gemcitabine is widely used in the treatment of pancreatic ductal adenocarcinoma (PDAC), but the development of chemoresistance poses a significant challenge to achieving long-term disease-free survival in PDAC patients.^1^ Resistance to gemcitabine may arise from various cellular and molecular changes in metabolism, tumor microenvironment, and DNA damage repair efficiency.^2^ Despite existing knowledge, large-scale molecular mechanisms underlying gemcitabine resistance remain unclear. Understanding these mechanisms could lead to more effective treatment strategies for this highly aggressive disease.

In this study, we created a CRISPR/cas9 sgRNA library targeting 791 human epigenome genes (Epi-library), with each gene being targeted by 6 different sgRNAs to minimize off-target effects. After introducing the library into PANC-1 cells, we treated them with either 2.5 μM or 25 μM of gemcitabine to identify genes associated with gemcitabine resistance through negative and positive selection processes (Fig. S1A). Our analysis revealed 15 significantly up-regulated and 29 down-regulated sgRNA sequences (Fig. 1A), with TULP4 being identified as a potential gemcitabine-resistant gene (Fig. S1B). In clinical observations, we found higher levels of TULP4 mRNA in pancreatic tissues compared with adjacent normal tissues (Fig. S1C), and patients who responded well to gemcitabine-based therapies generally had lower TULP4 expression levels compared with non-responders (Fig. 1B). Additionally, TULP4 expression significantly increased in tissues with disease progression (Fig. 1C, D), and higher TULP4 levels were associated with reduced overall survival rates (Fig. 1E). In our *in vitro* models of gemcitabine-resistant PANC1 and AsPC-1 cell lines (Fig. S2A), both mRNA and protein levels of TULP4 were all remarkably elevated (Fig. 1F, G). Manipulating TULP4 expression by overexpression or knockdown (Fig. S2B) resulted in altered sensitivity of PDAC cells to gemcitabine and affected their colony-forming abilities (Fig. 1H, I; Fig. S2C–E).

**Figure 1:**
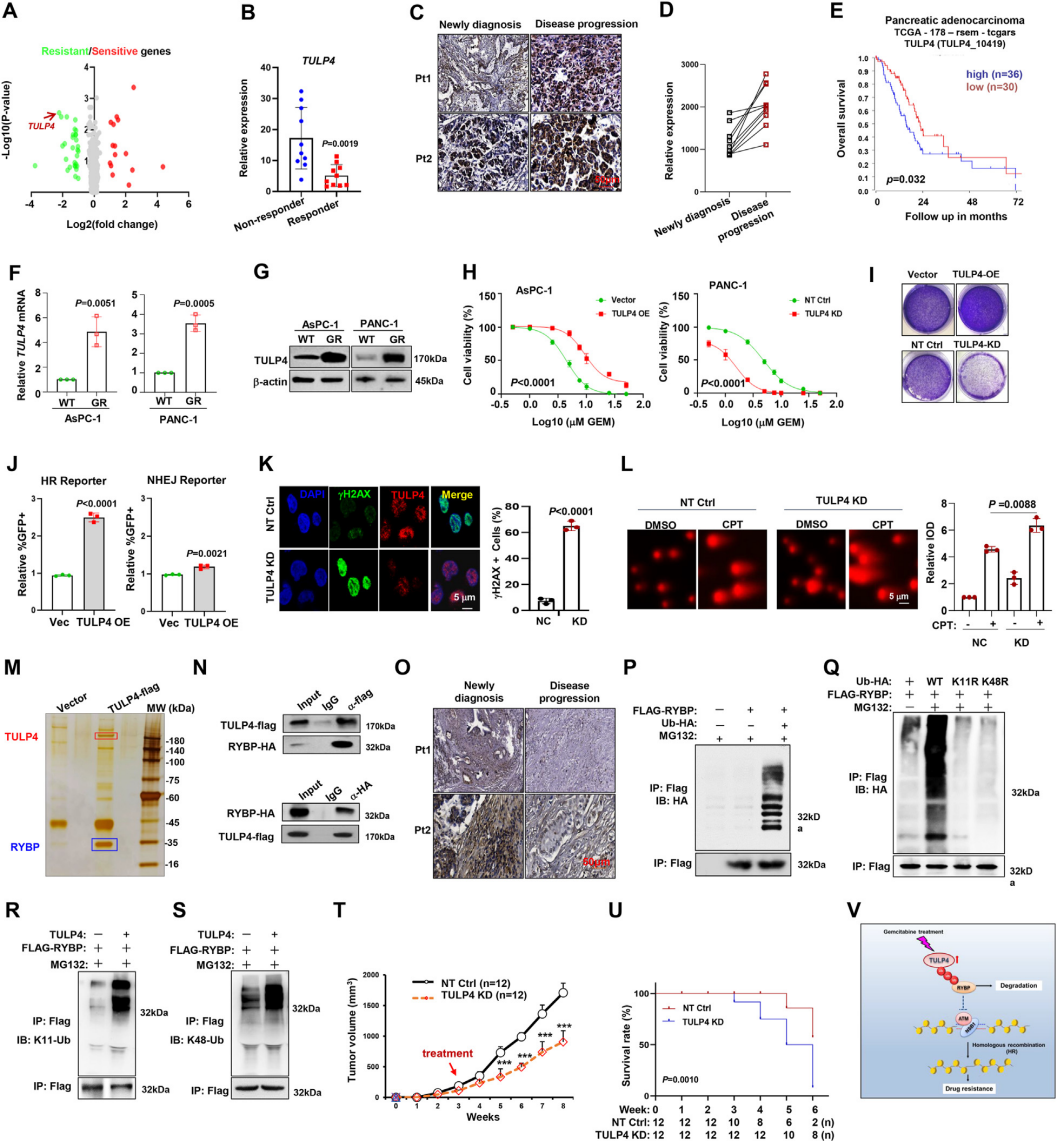
TULP4 mediates ubiquitination-dependent degradation of RYBP to enhance DNA damage repair and drug resistance in pancreatic ductal adenocarcinoma (PDAC). **(A)** The volcano plot illustrating the depleted genes in the negative selection and enriched genes in the positive selection. **(B)***TULP4* expression in patients who acquired complete response (CR, *n* = 10) or non-response (non-R, *n* = 10). **(C)** TULP4 level in immunohistochemical images in two patients with disease progression before and after treatment. Scale bar, 50 μm. **(D)** Expression trend of the *TULP4* gene before and after a gemcitabine-based treatment regimen. Seven patients with PDAC showed disease progression. **(E)** Correlation of *TULP4* mRNA expression with overall survival (OS) in TCGA database. **(F)** TULP4 mRNA expression and **(G)** protein level in WT and GR AsPC-1 and PANC-1 cells. **(H)** Alteration of IC_50_ to gemcitabine in Vehicle and TULP4-OE cells, or non-target control (NT Ctrl) and TULP-KD cells. **(I)** Colony formation of TULP4-OE and TULP4-KD PANC1 cells. **(J)** Quantitative assessment of homologous recombination (HR) and non-homologous end joining (NHEJ) activities in TULP4-OE and Vehicle AsPC-1 cells via flow cytometry detecting GFP^+^ cells among RFP^+^ cells. **(K)** Immunofluorescence assay for the level of γH2AX in TULP4-KD and NT Ctrl AsPC-1 cells treated with CPT (2 μM) for 4 h. Scale bar: 5 μm. **(L)** The comet assay images showing DNA damage in TULP4-KD PANC-1 cells treated with DMSO or CPT. Scale bar, 5 μm. **(M)** Silver staining for interactome of flag-TULP4 protein in PANC-1 cells. **(N)** Co-IP assay shows bilateral interactions between exogenous TULP4-flag and RYBP-HA in PDAC-1 cells. **(O)** RYBP level in immunohistochemical images in two patients at diagnosis and after disease progression. Scale bar, 50 μm. **(P)** Co-IP assay shows ubiquitination of RYBP in PANC-1 cells transfected with flag-RYBP and HA-ubiquitin with or without 10 μM MG132 for 6 h. **(Q)** Ubiquitination of flag-RYBP in PANC-1 cells transfected with negatively mutated HA-ubiquitin in the presence of 10 μM MG132 for 6 h. **(R)** Co-IP assay shows ubiquitination of RYBP in PANC-1 cells transfected with flag-RYBP and Ub-K11, **(S)** or Ub-K48 with 10 μM MG132 for 6 h. **(T)** Tumor growth of TULP4 KD or NT Ctrl gemcitabine-resistant (GR) PsAC-1 cells (3 × 10^6^ cells/mouse, *n* = 12) in nude mice. Gemcitabine (50 mg/kg) was given at week 3 after tumor inoculation for another 4 weeks. **(U)** The survival rate of nude mice at the time points of tumor diameter over 15 mm (*n* = 12). *p* values were determined by student's *t*-test for three independent experiments. **(V)** Treatment with gemcitabine stimulates the expression of E3 ligase TULP4, and TULP4 recruits and catalyzes polyubiquitination of the DNA repair repressor RYBP for degradation and consequentially prevents cell apoptosis and results in poor outcomes in the clinic. OE, overexpression; KD, knockdown; Co-IP, co-immunoprecipitation.

Gemcitabine primarily induces DNA damage, leading to growth arrest and cell apoptosis.^3^ We knocked down TULP4 expression in PANC-1 cells for bulk RNA sequencing and identified 173 down-regulated and 139 up-regulated genes (Fig. S3A). The Kyoto Encyclopedia of Genes and Genomes enrichment analysis revealed that differentially expressed genes were enriched in pathways related to DNA repair, drug resistance, apoptosis, and DNA replication (Fig. S3B). Our findings indicated that TULP4 plays a significant role in homologous recombination repair for DNA damage, as demonstrated by a flow cytometry-based reporter, consistent with our previous study^4^ (Fig. 1J). Additionally, the depletion of TULP4 compromised the DNA repair capacity of PDAC cells (Fig. S3C; Fig. 1K, L). Interestingly, levels of key members involved in homologous recombination repair pathways, such as p-ATM and p-P95/NBS1, were remarkably increased upon treatment with CPT (Fig. S3D).

To investigate the interaction between TULP4 and members of the interactome, a flag-tagged TULP4 protein was forcibly expressed in PANC1 cells for immunoprecipitation assay. The assay revealed that RecQ like helicase 4 (RYBP), a well-known suppressor of DNA replication and repair,^5^ interacted with TULP4 (Fig. 1M). Reciprocal co-immunoprecipitation assays and immunofluorescence staining further confirmed the interaction of TULP4 and RYBP, showing co-localized in the nucleus of PDAC cells (Fig. 1N; Fig. S4A). Observations in tissues from patients with disease progression showed a marked suppression of TULP4 and RYBP protein levels (Fig. 1O). As expected, RYBP protein was also down-regulated in gemcitabine-resistant PANC1 and AsPC-1 cells (Fig. S4B). Further investigation in gemcitabine-resistant PDAC cells revealed a significantly shortened half-life of RYBP (Fig. S4C, D). Subsequently, TULP4 overexpression compromised the stability of RYBP, whereas TULP4 knockdown hindered the degradation of RYBP (Fig. S4E–G). Analysis of major pathways for protein degradation unveiled that RYBP degradation was primarily proteasome-dependent (Fig. S5A), with RYBP being subject to poly-ubiquitination (Fig. 1P). Moreover, through modification of lysine residues of ubiquitin, we identified that poly-ubiquitination of RYBP protein was mainly through K11- and K48-linked ubiquitin (Fig. S5B; Fig. 1Q). Notably, overexpressing TULP4 in PANC1 cells resulted in RYBP protein degradation and facilitated K11-linked and K48-linked polyubiquitination of RYBP protein (Fig. S5C–E; Fig. 1R, S). In GR-PANC1 cells, a faster degradation of RYBP protein correlated with a higher ubiquitination level (Fig. S5F), and suppressing TULP4 reversed the ubiquitination of RYBP (Fig. S5G). Consequently, the E3 ligase TULP4 was identified as catalyzing RYBP polyubiquitination for degradation.

To investigate the effect of targeting TULP4 on gemcitabine resistance *in vivo*, we established a xenograft model using TULP4-knockdown or parental PANC-1 cells for gemcitabine treatment. Our findings showed that tumors derived from TULP4-knockdown cells exhibited significantly reduced growth rates, improved overall survival, higher levels of cell apoptosis, and increased γH2AX accumulation after gemcitabine treatment (Fig. 1T, U; Fig. S6A, B). Meanwhile, the protein level of RYBP was markedly rescued in the TUKP4-knockdown cell-derived tumors (Fig. S6C), accompanied by increased γH2AX level (Fig. S6D). Importantly, the expression of key genes related to tumor growth and survival, such as *Ki67* and *Bcl2*, were all meaningfully inhibited (Fig. S6E).

In conclusion, our study reveals that TULP4 is a gene associated with chemoresistance in PDAC cells. TULP4 disrupts RYBP protein to enhance DNA damage repair, thereby suppressing gemcitabine-induced cell apoptosis (Fig. 1V). Therefore, targeting the TULP4/RYBP axis could be a promising therapeutic strategy to overcome chemoresistance in PDAC patients.

## Ethics declaration

This study was approved by the Ethics Committee of the Fifth Central Hospital of Tianjin (No. TJWZXLL2022034). Informed consents were obtained from all patients.

## Author contributions

J.Y. Dai and S. Liu wrote the manuscript; J.Y. Dai, S. Liu, Y.X. Wang, Y.Y. Xie, T.T. Li, and L.C. Jia performed the experiments; J.Y. Dai and Z.G. Zhao analyzed the bioinformatics data; X.Y. Bian and T. Yu were in charge of the animal studies; Z.Q. Liu and X.Z. Liu approved the final version of this paper.

## Conflict of interests

The authors declared no conflict of interests.

## Funding

This study was funded by the 10.13039/501100007547Fourth Military Medical University Scientific Research Boost Fund (China) (No. YYKJFZJJ2018Y012), Tianjin Key Medical Discipline (Specialty) Construction Project (China) (No. TJYXZDXK-062B), the Tianjin Science and Technology Plan Project (China) (No. 22ZYQYSY00030), the Natural Science Foundation of Tianjin (No. 23JCZDJC00340), and Tianjin Health Technology Project (China) (No. TJWJ2022XK043).
