# Supplementary material for: TULP4 degrades RYBP to enhance DNA damage repair and chemosensitivity of pancreatic ductal adenocarcinoma
Source: Genes Dis. 2024 Apr 5;12(2):101288. doi: 10.1016/j.gendis.2024.101288 (PMC11605339; doi:10.1016/j.gendis.2024.101288)
Supplement: Multimedia component 1 [file mmc1.doc]

**Supplementary Data**

**Materials and Methods**

**PDAC cell lines**

Pancreatic ductal adenocarcinoma cells AsPC-1 and PANC-1 were from the National Infrastructure of Cell Line Resource (Beijing, China). PDAC cells were cultured in DMEM media supplemented with 10% of fetal bovine serum (Gibco, Life Technologies, Carlsbad, CA, USA). The HEK293T cells were cultured in DMEM-high glucose media with 10% fetal bovine serum (Gibco, Life Technologies, Carlsbad, CA, USA). These cells were all cultured at 37℃ in a humidified incubator with 5%CO2 (Gibco, Life Technologies, Carlsbad, CA, USA). All cells were STR authenticated (Biowing Biotech, Shanghai, China) and mycoplasma-free confirmed with the Universal Mycoplasma Detection Kit (ATCC, Manassas, VA, USA).

**Screening of genes using CRISPR/cas9 sgRNA system**

The human-specific epigenetic plasmid library (referred as Epi-library) was used to identify genes responsible for GEM resistance and sensitivity in PDAC cells. PANC-1 cells were transfected with the Epi-library at a low multiplicity of infection (MOI ≤ 0.3). After selected with 2 μg/ml of puromycin, at least 8 million transduced cells were treated with 25 μM GEM for 72h. After treatment, at least 3 replicate samples from each group were collected for genomic DNA extraction to ensure over 400× coverage of Epi-library. Genomic DNAs of samples were extracted using the FastPure Cell/Tissue DNA Isolation Mini Kit (Vazyme). The sgRNA cassettes were ampliﬁed using NEBNext® High-Fidelity 2× PCR Master Mix and next generation sequencing (NGS) was performed on an Illumina HiSeq to determine sgRNA abundance.

**Flow cytometry and cell viability assay**

Annexin V-FITC (FITC-conjugated Annexin V) (eBioscience, USA) was used to label apoptosis cells. Dead cells were labeled by PI (propidium iodide) (eBioscience, USA). Staining experiment was performed according to the product instructions. Briefly, one million cells were washed in cold PBS and suspended in 0.5 mL staining binding buffer. Annexin V-FITC (5 μL) and PI (5 μL) were added to the cell suspension respectively. Cells were incubated for 15 min at room temperature and subjected to flow analysis. The results were analyzed using FlowJo software. For CCK8 assays, PDAC cells were seeded at 1×10^5^ cells/well in triplicate in 96-well plates and were incubated at 37 °C with 5 % CO_2_. After incubation for 48 h, CKK8 reagent was added to each well and incubated for 2 h prior to reading absorbance at 450 nm. The following formula was used to calculate cell viability (%) = OD value of treatment group/OD value of control group ×100.

**Quantitative Real-Time PCR(Q-PCR)**

Total RNA was extracted with the assistance of TRIzol reagent (Takara). and reverse transcribed using the SuperScript III RT kit and oligo dT primers (Invitrogen, Carlsbad, CA). PCR primers were purchased from Integrated DNA Technologies (Coralville, IA). Real-time quantitative PCRs (qPCR) were performed using iTaqTM Universal SYBR® Green Supermix (Bio-Rad, USA). Fold changes were calculated using the ΔΔC_t_ method and GAPDH message as reference.

**Western blotting and Co-immunoprecipitation (Co-IP)**

Western blots were performed to detect expression levels of proteins in PDAC cells. Briefly, cells were lysed on the ice and then total lysate (10 μg) was fractionated by SDS-PAGE in 4%-12% polyacrylamide gels. Proteins were transferred to PVDF to nitrocellulose. After blocked with 5% non-fat dry milk, membranes were incubated overnight at 4°C with primary antibodies. Protein bands were visualized with HRP-conjugated secondary antibody using Super Signal West Pico (Pierce) and evaluated by densitometry to estimate protein abundance. Co-IPs using the Pierce™ Direct Magnetic IP/Co-IP kit (Thermo Scientific) and antibodies to anti-TULP4 (NOVUS, NBP1-92546) and anti-FLAG M2 Affinity Gel (Sigma-Aldrich, St. Louis, MO, USA), anti-RYBP (abcam, ab5976). IgG from Bethyl Laboratories was used as control.

Briefly, total proteins from TULP4 overexpressing AsPC-1 cells were extracted with IP lysis buffer. FLAG antibodies or control immunoglobulin (IgG) were added and vertical rotated with cell lysate overnight at 4 °C. Then protein A/G magnetic beads was added into the protein-antibody complex and incubated overnight at 4 °C. The beads were washed three times with IP lysis buffer. The pulled-down proteins were extracted and detected by Western blotting as described above.

**Immunohistochemistry**

Tissue samples were formalin-fixed and paraffin-embedded. Slides were deparaffinized and rehydrated via successive immersion in the following solutions: 100% xylene I (10 min), 100% xylene II (10 min), 100% ethanol I (5 min), 100% ethanol II (5 min), 95% ethanol (5 min), 85% ethanol (5 min), 80% ethanol (5 min), 75% ethanol (5 min), 80% ethanol (5 min), double distilled water I (10 min), and double-distilled water II (10 min). The slides were then boiled in 0.01 mol/L citrate buffer at 99 °C for 20 min, and endogenous peroxidase activity was blocked with 0.3% H_2_O_2_ in methanol for 30 min. Goat serum was used to block the antibody at room temperature for 10 min. After overnight incubation at 4 °C in primary antibodies, the slides were exposed to HRP-labeled secondary antibodies for 1 h at room temperature and developed with 3,3′-diaminobenzidine system. Staining intensity was accessed by a designated member of our group using the Image-pro Plus 6.0 (Media Cybernetics) and was represented by the mean density, using the formula mean density = integrated optical density/area of interest.

**Immunofluorescence staining**

Cells (1×10^5^) were spun down on glass slides, washed and then fixed with 4% paraformaldehyde solution (Affymetrix, USA) for 15 min at room temperature. After permeabilized and blocked, the cells were exposed to anti-γH2AX antibody (1:100) (millipore, 05-636, Darmstadt, Germany) and TULP4 antibodies overnight at 4 °C. The cells were incubated with secondary antibodies coupled to Alexa-Fluor® 488 goat anti-rabbit IgG(H+L) or Alexa-Fluor® 594 goat anti-mouse IgG(H+L) for 1 h at room temperature in the dark. Nuclei were counterstained with DAPI 4’,6-diamidino-2-phenylindole (DAPI). Fluorescence was visualized on a fluorescence microscope (FV-1000, OLYMPUS).

**Mass spectrometry**

Cells were resuspended in NP-40 lysis buffer (150mM sodium chloride, 1% NP-40, 50 mM Tris PH 8.0) with protease inhibitor cocktail (Roche) and centrifuged at 12 000 g for 20 min at 4°C. The supernatants were incubated with anti-FLAG M2 affinity gel at 4°C overnight. After washing four times with NP-40 lysis buffer, FLAG protein complex was eluted with FLAG peptide (Sigma). The elutes were resolved on NuPAGE 4%-12% Bis-Tris gel (Invitrogen) and stained with a silver staining kit (Pierce). The protein bands were cut out and analyzed by liquid chromatography-tandem mass spectrometry (LC-MS/MS).

**NHEJ Assay and HR Assay**

A total of 2×10^6^ PsAC-1 cells transduced with vector, TULP4 OE or Vector were transfected with 1 μg of eGFP-NHEJ/HR reporter with RFP vector as control. Cells were harvested 48 h later and assayed with flow cytometry system (BD Bioscience) for GFP expression.

**Laser micro-irradiation**

Laser micro-irradiation was performed in WT and GEM resistant (GR) PsAC-1 cells using the PLAM Micro Beam (ZEISS, oberkochen, German). The laser output was set to 45%, which can reproducibly give a focused γH2AX stripe. Then, the samples were subjected to immunofluorescence staining with indicated antibodies.

**Xenograft model in nude mouse**

Animal studies were approved by the Committee on Animal Research and Ethics of Tianjin Medical University, and all protocols were confirmed to the Guidelines for Ethical Conduct in the Care and Use of Nonhuman Animals in Research. PsAC-1 TULP4 KD cells and NT Ctrl cells (1.5 × 10^6^) were injected subcutaneously in the abdominal area of 4-6 weeks old nude mice (SPF Beijing Biotechnology, Beijing, China). Beginning on day 7 post cell transfer, mice were treated with GEM or PBS as control twice weekly until humane endpoints were reached. The tumors volume was measured every 3 days and calculated by (length × width^2^)/2. All animal experiments were conducted in compliance with the protocol approved by the Institutional Animal Care and Use Committee of Tianjin Medical University.

**Tunnel assay**

Tunnel assay was performed according to DeadEnd™ Fluorometric TUNEL System (Promaga, Tokyo, Japan) manufacturer’s instructions. Briefly, tumor sections were treated with fresh xylene in a Coplin jar for 5 min twice at room temperature. Rehydrate the samples by sequentially immersing the slides through graded washes (100%, 95%, 85%, 70%, 50%, 0.85% NaCl, PBS) for 5 min. The tissue sections were fixed by immersing the slides in 4% methanol-free formaldehyde solution in PBS for 15 min. After washed in PBS for 5 min three times, the samples were incubated with 100µl proteinase K (20µg/ml) for 8-10 min. Then were washed and incubated with 100µl Equilibration Buffer for 5-10 min. The tissue sections were fixed with 4% methanol-free formaldehyde solution in PBS for 5 min. Then washed and incubated with 50µl of rTdT incubation buffer at 37°C for 60 min in the dark. The reactions were terminated by incubated with 2× SSC for 15 min at room temperature. The samples were washed three times and were stained by propidium (1µg/ml) in PBS for 15 min at room temperature in the dark. Then the samples were washed three times and analyzed by the Olympus FV1000 IX81-SIM Confocal Microscope (Olympus, Tokyo, Japan).

**Statistical analysis**

Data were shown as mean ± SD for at least three independent experiments. Differences between groups were determined using paired two-tailed Student’s t-test or two-way ANOVA. Comparison of Kaplan-Meier survival curves was performed by Log-rank (Mantel-Cox) test, Pearson correlation test was used to determine the correlations between gene expressions, and survival analysis and a log-rank test was done. A *P* value less than 0.05 was considered statistically significant.

**Supplementary Figures**

**Figure S1**


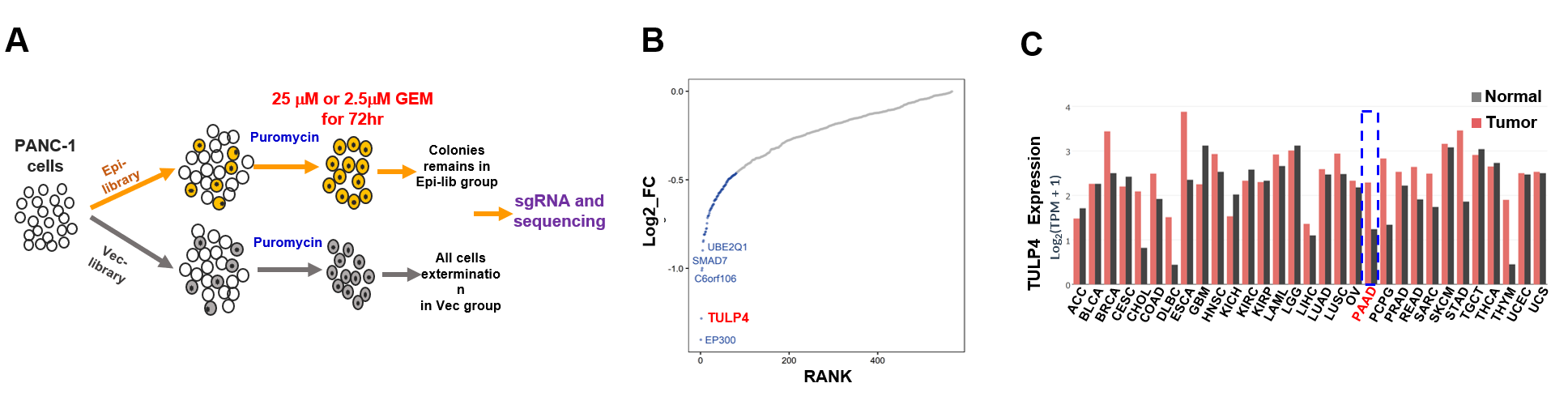


**Fig. S1** **CRISPR/cas9 sgRNA screening identifies TULP4 as a drug-resistant gene**

**(A)** Diagram of epigenome-wide CRISPR/cas9 screening system in PANC-1 cells. The cells selected by puromycin and GEM were chosen for screening. (**B**) Illustration of the top 5 gene candidates from the above screening. (**C**) Comparison of *TULP4* expression in tumors and adjacent normal tissues of31 categories of tumors from the GEPIA database.

**Figure S2**


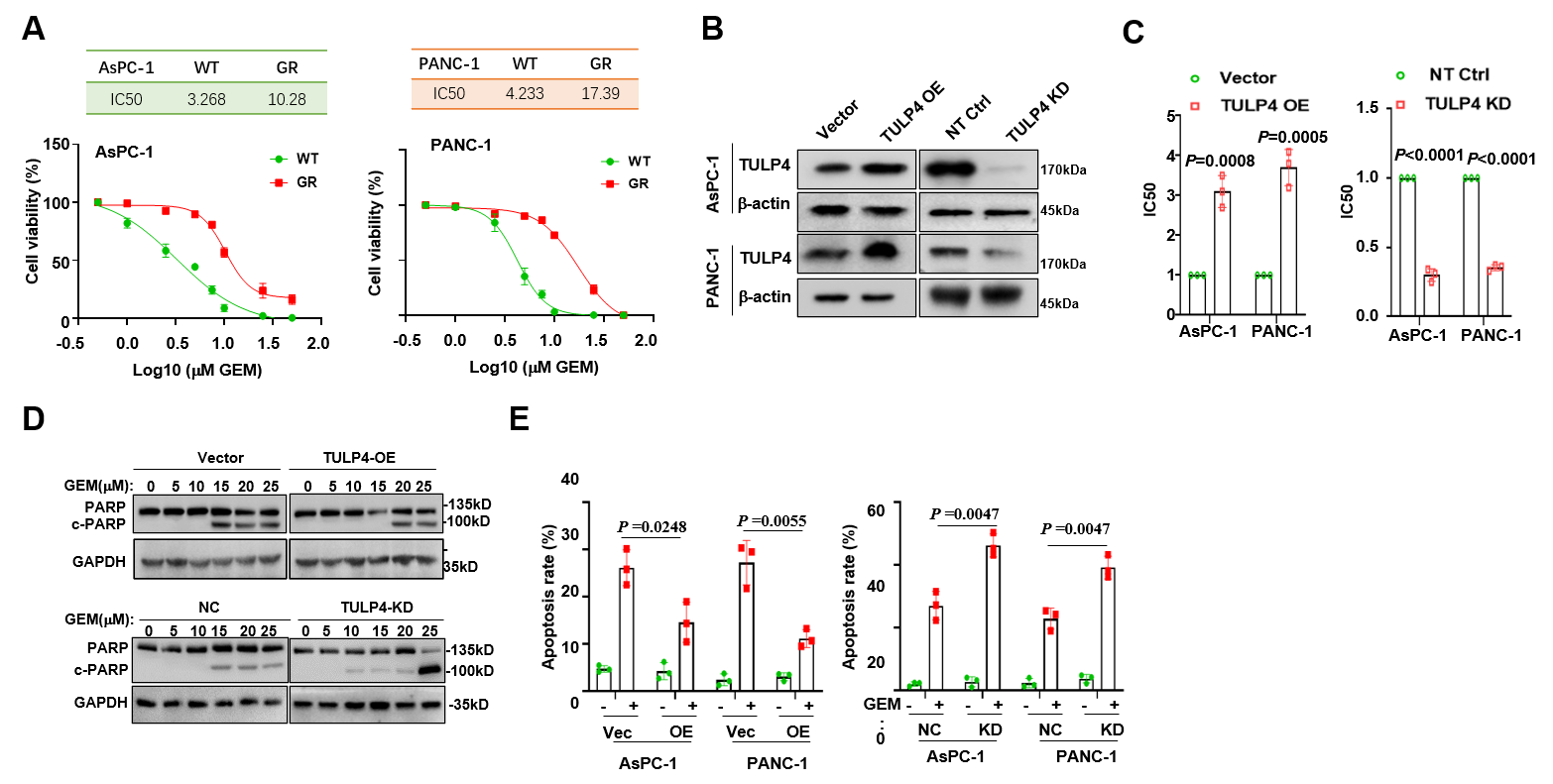


**Fig. S2 TULP4 is upregulated in GEM-resistant pancreatic cancer cell**

**(A)** Alteration of IC_50_ to gemcitabine in the wild type (WT) and GEM resistant (GR) AsPC-1 and PANC-1 cells. (**B**) Western blotting shows the efficacy of TULP4 knockdown and overexpression in AsPC-1 and PANC-1 cells. (**C**) Comparison of IC_50_ to GEM in Vector and TULP4 OE cells, or NT Ctrl and TULP4-KD AsPC-1 and PANC-1 cells (n = 3). **(D)** Cleavage of PARP in Vehicle and TULP4-OE, or NT Ctrl and TULP4-KD AsPC-1-GR cells treated with GEM for 48 hours (n = 3). (**E**) Flow cytometry analysis for apoptosis of Vehicle and TULP4-OE cells, or NT Ctrl and TULP4-KD AsPC-1 and PANC-1 cells treated with GEM (5 μM) for 48 h. *P* value determined by Student’s t test for n = 3 independent experiments.

**Figure S3**


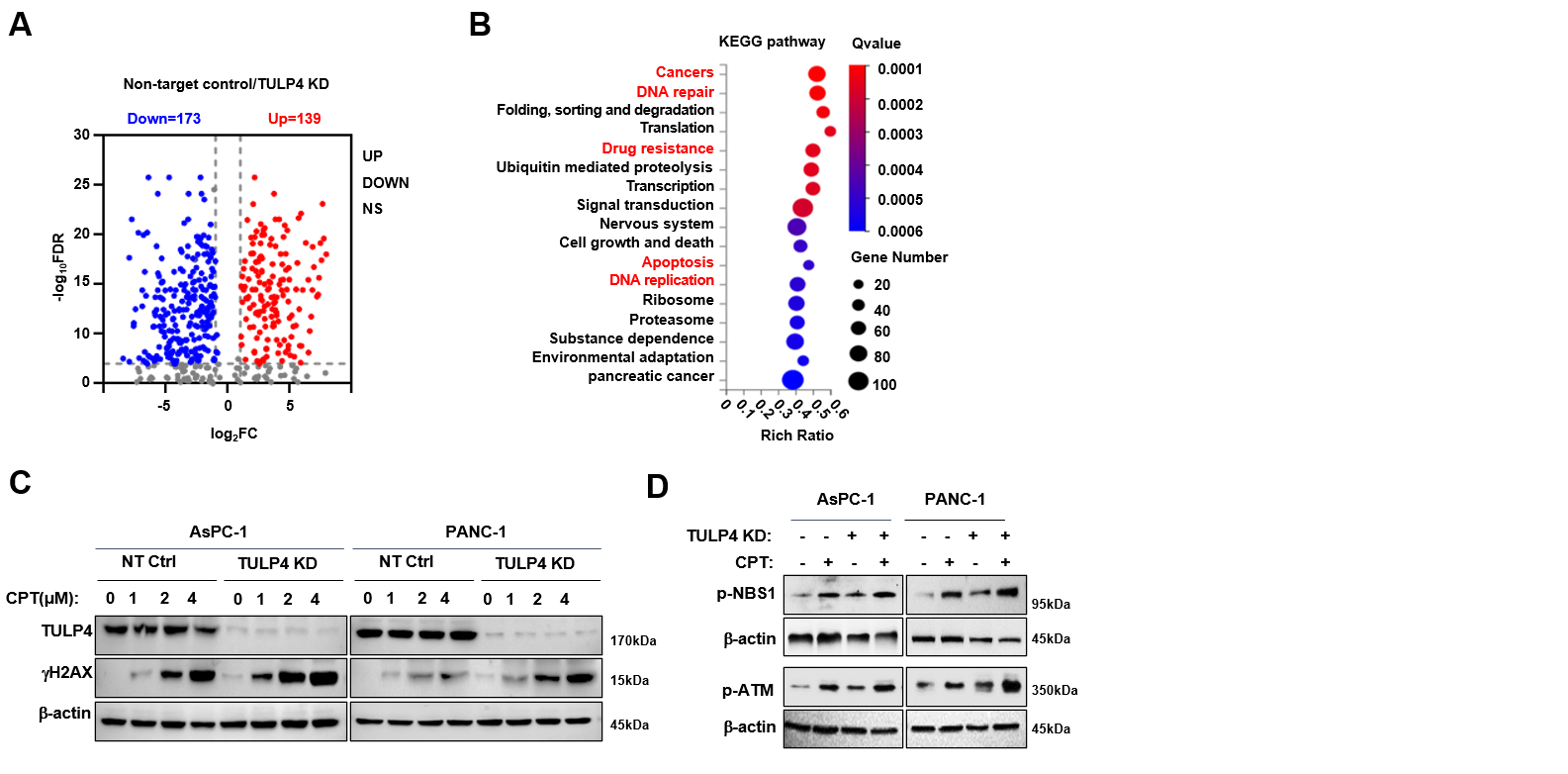


**Fig. S3 TULP4 facilitates homologous recombination to repair DNA damage**

**(A)** Volcano plot of differentially expressed genes analyzed from bulk RNA-sequencing in TULP4-KD AsPC-1 cells. Blue, downregulated genes; red, upregulated genes; gray, statistically non-significance genes.  **(B)** KEGG analysis for differentially expressed genes with a *P* < 0.05 using DAVID methods.  **(C)** Western blotting shows γH2AX in NT Ctrl and TULP4-KD AsPC-1 and PANC-1 cells treated with CPT for 24 h. **(D)** Western blotting shows p-NBS1 and p-ATM levels in NT Ctrl and TULP4-KD AsPC-1 and PANC-1 cells.

**Figure S4**


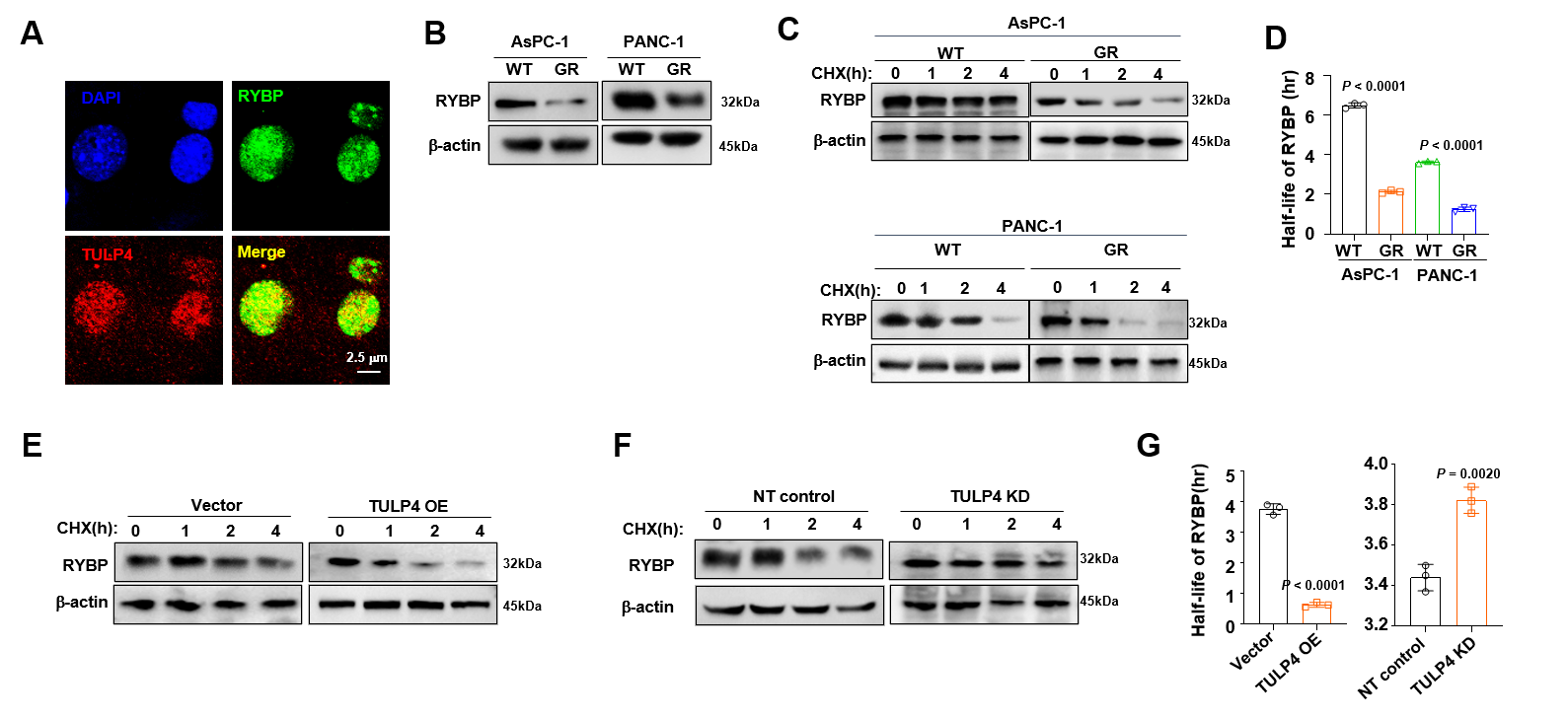


**Fig. S4 TULP4 modulates the stabilization of RYBP**

**(A)** Immunofluorescence staining to show co-localization of TULP4 and RYBP in PANC-1 cells. **(B)** Western blotting assay shows the RYBP protein level in the WT and GR AsPc-1 and PANC-1 cells. **(C)** Degradation rate of RYBP protein in WT and GR AsPC-1 and PANC-1 cells treated with 20 μM cycloheximide (CHX) for different times, and (**D**) shows the calculated half-life. Degradation rate of RYBP protein in **(E)**TULP4-OE, or **(F)** TULP4-KD PANC-1 cells treated with 20 μM cycloheximide (CHX) for different times, and **(G)** shows the calculated half-life of RYBP protein of (E) and (F).

**Figure S5**


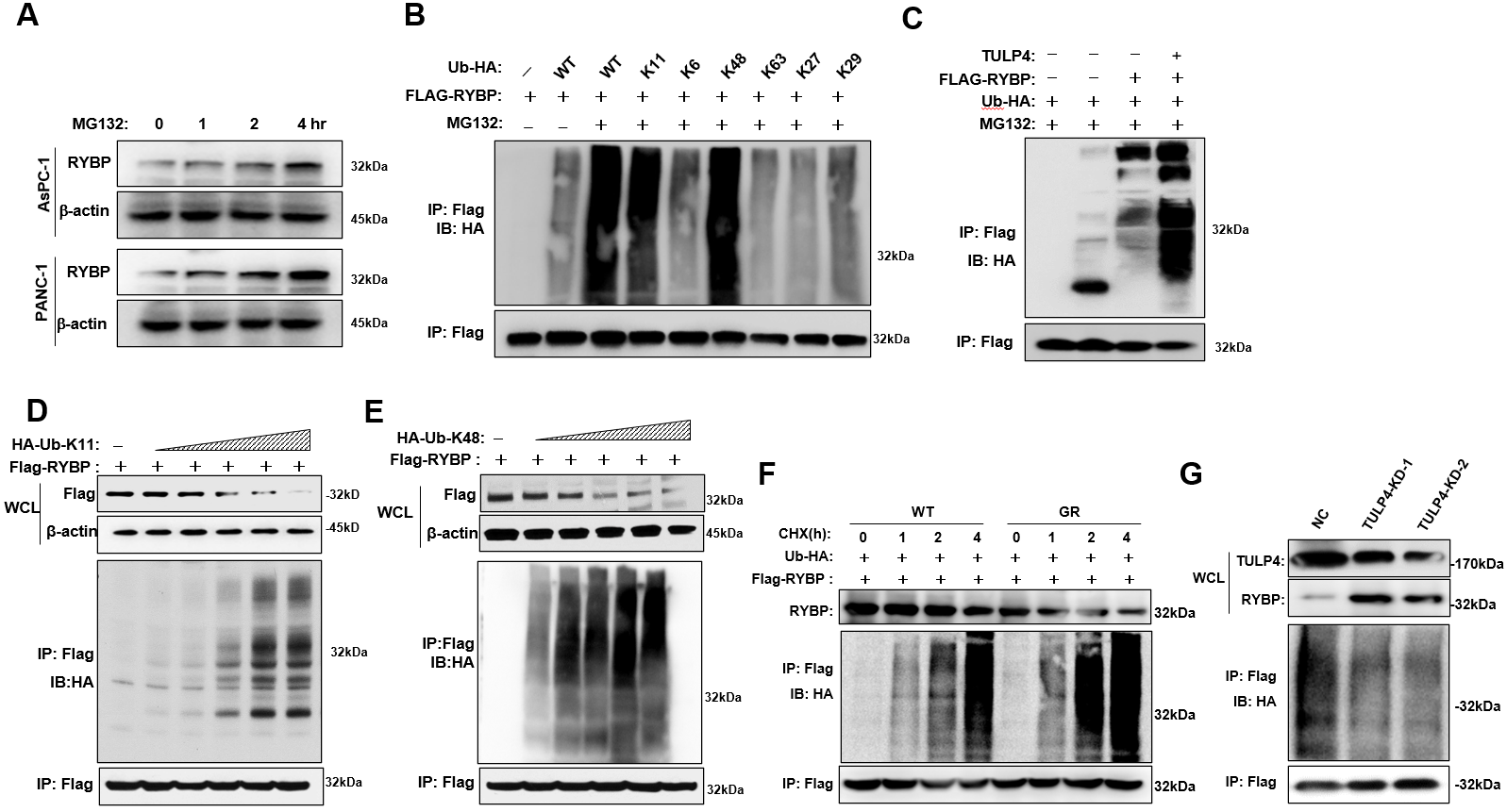


**Fig. S5 TULP4 alleviates RYBP ubiquitination**

(**A**) Western blotting assay shows the RYBP protein level in AsPC-1 and PANC-1 cells treated with 10μM MG132 for different times. **(B)** Mapping the key lysine residues of poly-ubiquitination on TULP4 proteins in PANC-1 cells transfected with flag- RYBP or a series of positively mutated HA-ubiquitin in the presence of 10 μM MG132 for 6 hours. **(C)** Western blotting assay shows the endogenous RYBP in PANC-1 cells transfected with TULP4 overexpressing vector for 48 hr. Flag- RYBP levels in PANC-1 cells co-transfected with Ub-K11**(D)**, or **(E)** Ub-K48 for 48 hr and treated with MG132 (10 mM) for 4 hr before harvesting proteins were detected by anti-flag IB. **(E) (F)** Ubiquitination of flag-RYBP in the WT and GR PANC-1 cells treated with 20μM CHX for different time. **(G)** Ubiquitination of flag-RYBP in PANC-1 cells with TULP4 knock down with 2 different shRNAs.

**Figure S6**


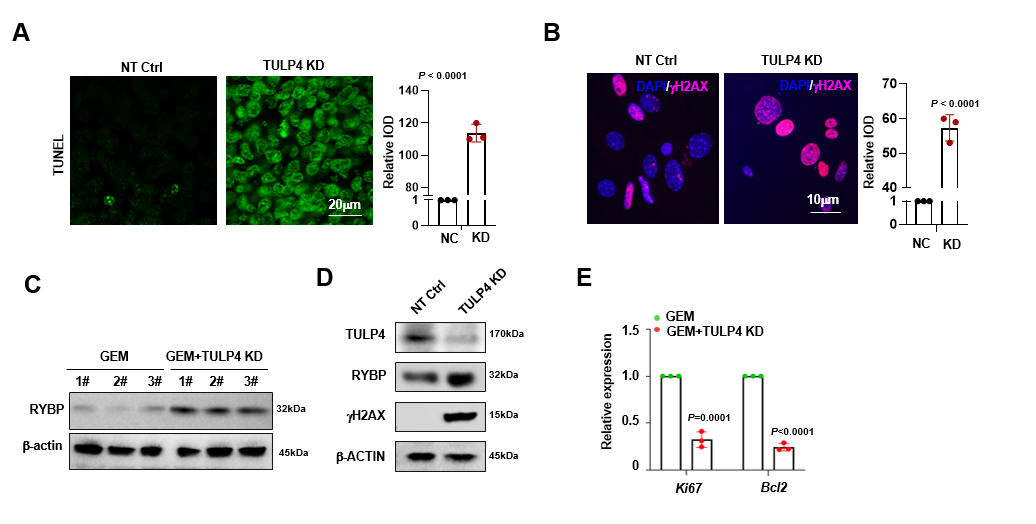


**Fig. S6 TULP4 knockdown inhibits tumor formation and improves GEM sensitivity in *vivo***

**(A)** TUNEL assay for tissues from xenograft of TULP4-KD or NT Ctrl cells treated with GEM (50 mg/kg). Scale Bar: 5 μm **(B)** Immunofluorescence assay showing the level of γH2AX in tissues from xenograft derived from NT Ctrl or TULP4-KD PANC-1 cells and treated with GEM (50 mg/kg). Scale Bar: 10 μm. **(C)** Protein levels of RYBP in the xenograft tissue of different treatment groups. **(D)** Correlation of RYBP and γH2AX in the xenograft tissue of NT Ctrl and TULP4-KD PANC-1 derived tumors.  **(E)** *Ki67* and *Bcl2* mRNA expression in NT Ctrl and TULP4-KD tumor tissues. *P* value determined by Student’s t test for n=3 independent experiments.
